# Minimally Invasive Syringe‐Injectable Hydrogel with Angiogenic Factors for Ischemic Stroke Treatment

**DOI:** 10.1002/adhm.202403119

**Published:** 2024-11-09

**Authors:** Donggue Kim, Ji Woo Lee, Yang Tae Kim, Junhyeok Choe, Gaeun Kim, Chang Man Ha, Jae Geun Kim, Kwang Hoon Song, Sunggu Yang

**Affiliations:** ^1^ Department of Nano‐Bioengineering Incheon National University Incheon 22012 Republic of Korea; ^2^ Division of Life Sciences College of Life Sciences and Bioengineering Incheon National University Incheon 22012 Republic of Korea; ^3^ Research Division and Brain Research Core Facilities of Korea Brain Research Institute Daegu 41068 Republic of Korea; ^4^ Research Center of Brain‐Machine Interface Incheon National University Incheon 22012 Republic of Korea; ^5^ gBrain Inc. Incheon 21984 Republic of Korea

**Keywords:** angiogenesis, electrospinning, gelatin‐norbornene, ischemic stroke, sensorimotor function

## Abstract

Ischemic stroke (IS) accounts for most stroke incidents and causes intractable damage to brain tissue. This condition manifests as diverse aftereffects, such as motor impairment, emotional disturbances, and dementia. However, a fundamental approach to curing IS remains unclear. This study proposes a novel approach for treating IS by employing minimally invasive and injectable jammed gelatin‐norbornene nanofibrous hydrogels (GNF) infused with growth factors (GFs). The developed GNF/GF hydrogels are administered to the motor cortex of a rat IS model to evaluate their therapeutic effects on IS‐induced motor dysfunction. GNFs mimic a natural fibrous extracellular matrix architecture and can be precisely injected into a targeted brain area. The syringe‐injectable jammed nanofibrous hydrogel system increased angiogenesis, inflammation, and sensorimotor function in the IS‐affected brain. For clinical applications, the biocompatible GNF hydrogel has the potential to efficiently load disease‐specific drugs, enabling targeted therapy for treating a wide range of neurological diseases.

## Graphical abstract

The developed GNF/GF are administered to the motor cortex of IS models to evaluate their therapeutic effects on stroke‐induced motor dysfunction. GNFs mimic a natural fibrous extracellular matrix architecture and can be precisely injected into a targeted brain area. The GNF/GF system increased angiogenesis and sensorimotor function in the IS models.

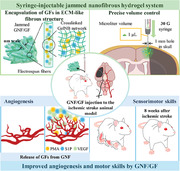

## 1 Introduction

Ischemic stroke (IS), which constitutes the majority of stroke cases, occurs mainly because of the blockage of blood vessels toward the brain, leading to the depletion of oxygen and glucose, and subsequent cell death.^[^ ^1^ ^]^ This shortage of blood supply to the brain can eventually lead to various aftereffects, such as sensorimotor impairment, speech and communication deficits, memory impairment, emotion processing deficits, and mental disorders. In particular, ≈50% of survivors of IS suffer from sensorimotor deficits.^[^ ^2^ ^]^ Furthermore, IS often accompanied by various complications, such as cardiac complications, pneumonia, venous thromboembolism, fever, pain, dysphagia, incontinence, and depression, all of which affect patients’ daily lives and impede neurological recovery.^[^ ^3^ ^]^ Currently, the clinical treatment of IS primarily involves the restoration of blood flow to the brain. For example, intravenous thrombolytics are commonly used as IS treatments to dissolve clots that block blood vessels. An endovascular procedure using catheters and stents to physically remove blood clots is an alternative approach.^[^ ^4^ ^]^ However, conventional approaches require swift action following the onset of stroke and merely offer temporary solutions with a high risk of recurrence. In addition, they may not be able to support the regeneration of brain tissues already damaged by stroke. Therefore, there is a growing need to develop an effective IS treatment that can fundamentally restore the damaged brain following its aftereffects.^[^ ^1^, ^2^ ^]^

Pharmacological approaches are the current mainstay treatments for IS,^[^ ^5^, ^6^ ^]^ and various strategies have been developed to deliver appropriate drugs to target brain tissues. Drugs injected into the bloodstream may not be able to approach the target brain tissues because the blood–brain barrier (BBB) hinders the extravasation of drugs.^[^ ^7^ ^]^ Advances in drug delivery systems, such as receptor‐mediated transcytosis, lipophilic pathways, and paracellular pathways, have allowed the penetration of drugs through the BBB and, eventually, the treatment of IS.^[^ ^8^, ^9^, ^10^ ^]^ However, even if drugs cross the BBB, they may not directly reach the infarct area. Since the infarct area is directly related to blocked blood vessels, drugs administered through the bloodstream may not effectively reach the affected region. Alternatively, drug‐carrying vehicles can be directly injected into brain tissues, and the release of the loaded drugs effectively treats IS. For example, drug‐loaded hydrogels were injected into the brain tissues of IS models for IS treatment.^[^ ^11^, ^12^, ^13^, ^14^ ^]^

Hydrogels that mimic various features of the extracellular matrix (ECM) have been developed as drug‐delivery vehicles,^[^ ^15^ ^]^ and injectable drug‐delivery hydrogels were further developed for various biomedical applications.^[^ ^16^ ^]^ Jammed hydrogels, developed as injectable hydrogels, were injected into sciatic nerves^[^ ^17^ ^]^ and diabetic wounds^[^ ^18^ ^]^ to deliver brain‐derived neurotrophic factor and small interfering ribonucleic acid, respectively. Both in situ cross‐linking hydrogels and suspended granular hydrogels have been used as injectable hydrogels targeting specific brain regions in several previous studies (**Table** **1**). An adjustment of crosslinking duration and timing for the precursors is required to control the viscosity and maximize the drug‐delivery efficiency of *in‐situ* crosslinking hydrogels. Suspending granular hydrogels, a mixture of phosphate‐buffered saline (PBS), and hydrogels at the microscale is also effective for drug delivery. However, it is difficult to control the overall volume of hydrogels injected in a suspension state because they can leave the target site, resulting in a distribution of the granular hydrogels that reduces the effectiveness of the therapy.^[^ ^19^, ^20^ ^]^

**Table 1:** Comparison of our jammed fibrous hydrogel with *in‐situ* injectable and granular hydrogels targeting brain tissue. Simple injection information for both systems, such as the main polymer, injected volume, and diameter of the needle.

Injected material		Structure (size)	Main polymer	Injected volume [µL]	Needle [gauge (G)]	References
Fibrous hydrogel	Jammed pellet	Nanofiber (800 nm)	GelNBa	2	30	This study
In situ hydrogel	Precursor	Bulk	HT^a)^	20	Micro‐syringe	[46]
			PEGA^b)^	3	Micro‐syringe	[47]
			PC^c)^	10	23	[48]
Granular hydrogel	Gel suspension	Round particles (150 µm)	PEGDA^d)^	10	Micro‐syringe	[49]
		Round particles (30 µm)	Gelatin	5	Micro‐syringe	[50]
		Round particles (146 µm)	GelMA^e)^	5	31	[51]

^a)^ Tyramine‐hyaluronic acid;

^b)^ PEG acrylate;

^c)^ Phenol‐chitosan;

^d)^ PEG diacrylate;

^e)^ Gelatin‐methacrylate.

In this study, we developed an injectable jammed nanofibrous hydrogel that overcomes the aforementioned concerns to facilitate angiogenesis in an IS animal model with a middle cerebral artery occlusion (MCAO). Our injectable hydrogels carrying growth factors (GFs; including vascular endothelial growth factors (VEGF), sphingosine 1‐phosphate (S1P), and phorbol 12‐myristate 13‐acetate (PMA)) are expected to induce rapid angiogenesis, resulting in the growth of many new blood vessels. In addition to VEGF, which promotes the growth of new blood vessels by stimulating endothelial cell proliferation and migration,^[^ ^21^ ^]^ the concentration of S1P is known to influence the speed of endothelial cell invasion and the multicellularity of sprouts,^[^ ^22^, ^23^ ^]^ while PMA is known to increase the diameter of angiogenic sprouts.^[^ ^23^ ^]^ After injection of the GFs into the IS brain, angiogenesis and sensorimotor function were significantly restored. These data suggest that our injectable jammed hydrogels have the potential to effectively deliver therapeutics and target specific areas of various diseased brains. To the best of our knowledge, this is the first study to develop an injectable jammed nanofibrous hydrogel carrying GFs for tissue targeting. This system enables the precise injection of cell‐friendly nanofibrous hydrogels using ultrathin needles. In future studies, we plan to explore the potential of this system by fabricating various hydrogels.

## 2 Results

**Figure 1:**
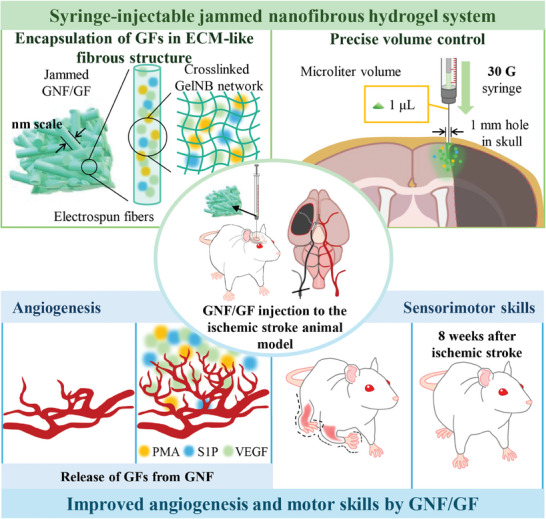
Schematic illustration of the experiments. In an animal IS model, the injection of a GNF/GF hydrogel (with a structure similar to the fibrous ECM) into the motor cortex enhanced angiogenesis and sensorimotor function.

To investigate the therapeutic effects of hydrogels of gelatin modified with norbornene (GelNB) nanofibers (GNF group) and those carrying GFs (GNF/GF group), on IS‐induced sensorimotor dysfunction, we injected the jammed GNF/GF system into the motor cortex of IS rats. The GNFs provide a similar fibrous structure as found in the ECM. The resulting GNF‐based hydrogels are syringe injectable and drug delivery can be achieved through a 1‐mm hole in the skull. In the IS animal model, the injection of GNF/GF into the motor cortex promoted angiogenesis and sensorimotor function (**Figure** **1**).

### 2.1 Fabrication of Jammed Nanofibrous Hydrogels to Simulate the ECM

**Figure 2:**
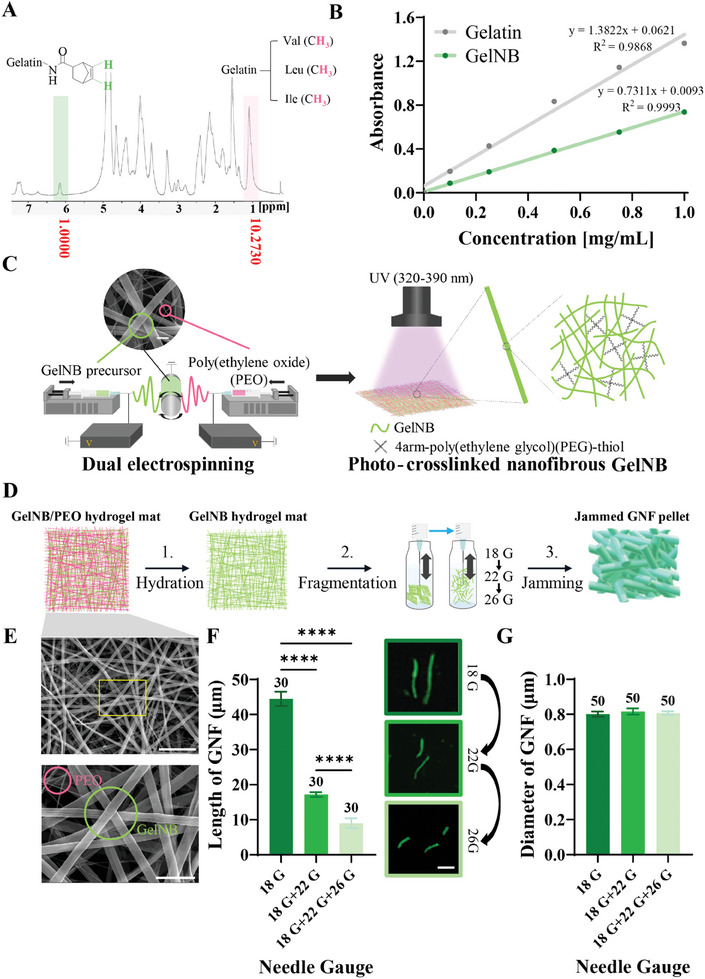
Overall process to fabricate injectable nanofibrous GelNB hydrogels. Degree of substitution of GelNB measured by A) ^1^H NMR and B) TNBSA assay. The obtained NMR peaks were analyzed by comparing the norbornene double bond signals (left, green) with the methyl proton signals (right, pink). The TNBSA assay was performed by comparing the slopes of the equations for Gelatin and GelNB. C) Illustration of dual electrospinning and photocrosslinking processes. The GelNB and PEO precursors were electrospun at a ratio of 4:6 and then exposed to UV light to photocrosslink the GelNB nanofibers (PEO nanofibers in the GelNB/PEO mat were not crosslinked). D) Illustration of the fabrication process after dual electrospinning. The photocrosslinked fibrous GelNB/PEO hydrogel mats were hydrated in PBS to remove the sacrificial PEO nanofibers. Then, pure GelNB hydrogel mats were fragmented by repeated needle pumping from 18 G to 26 G. The GNF solution was centrifuged to obtain jammed GNF pellet. E) Representative SEM images of the dual electrospun GelNB/PEO. The thicker fibers are GelNB and the thinner fibers are PEO. The yellow box represents the region where the magnified image below was obtained (×10). Scale bar: 10 µm (above), 3 µm (below). F) Length of GNFs at each step of needle pumping. Needles with a smaller inner diameter gave shorter GNFs. Scale bar: 10 µm (*n* = 30, p < 0.005). G) Diameter of the GNFs at each step of needle pumping. The fragmentation steps did not affect the diameter of the GNFs, which were all ≈800 nm (*n* = 50, p = 0.77). The numbers above each bar give the sample size (*n*) for each group. p values were determined by one‐way analysis ANOVA with Bonferroni's post‐hoc test in the three groups (needle gauge). All data are presented as the mean ± the standard error of the mean (SEM). *p< 0.1, **p< 0.05, ***p< 0.01, ****p< 0.005.

The fibrous hydrogels obtained by electrospinning were dissected into small pieces and jammed via centrifugation. Gelatin was modified with norbornene to form the GelNB hydrogels. The norbornene content in the GelNB was ≈46.9 % and 47.1 %, as determined by nuclear magnetic resonance (^1^H NMR) (**Figure** **2**A) and 2,4,6‐trinitrobenzene sulfonic acid (TNBSA) (Figure 2B) assays, respectively. A poly(ethylene oxide) (PEO) precursor was electrospun to generate dehydrated PEO fibers, which were deposited as a sacrificial layer to enable the fibers to be easily peeled off the collector. PEO and GelNB precursors (4arm‐poly (ethylene glycol) (PEG)‐thiol and 2‐hydroxy‐4'‐(2‐hydroxyethoxy)‐2‐methylpropiophenone (Irgacure 2959) dissolved in water) were simultaneously electrospun to obtain dehydrated GNFs interwoven with dehydrated PEO nanofibers. This process was followed by illuminating the interwoven fibers with ultraviolet (UV) light to photopolymerize the GNF (Figure 2C). The interwoven fibers were then hydrated in PBS to remove the PEO fibers and increase the porosity of the GNF mat (Figure 2D). The hydrated GNF were then fragmented by sequential pumping through ascending size of needles of 18, 22, and 26 gauge (G) and then jammed by centrifugation. The dehydrated interwoven fibers before PBS hydration were visualized using scanning electron microscopy (SEM) (Figure 2E). The pumping of the GNF decreased the length of the fragmented fibers (18 G: 44.46 ± 11.12 µm, *n* = 30; 18 G + 22 G: 17.18 ± 3.77 µm, *n* = 30; 18 G + 22 G + 26 G: 9.03 ± 1.42 µm, *n* = 30; one‐way ANOVA with Bonferroni's test; Figure 2F) but did not change their diameter significantly (18 G: 0.80 ± 0.015 µm, *n* = 50; 18 G + 22 G: 0.82 ± 0.017 µm, *n* = 50; 18 G + 22 G + 26 G: 0.81 ± 0.11 µm, *n* = 50; one‐way ANOVA with Bonferroni's; Figure 2G). The length and diameter of GNF were similar to those of collagen fibers investigated in a previous study of the ECM (length: ≈3.6–21.9 µm, diameter: ≈0.310–0.899 µm).^[^ ^24^ ^]^

### 2.2 Injectability of GNF

**Figure 3:**
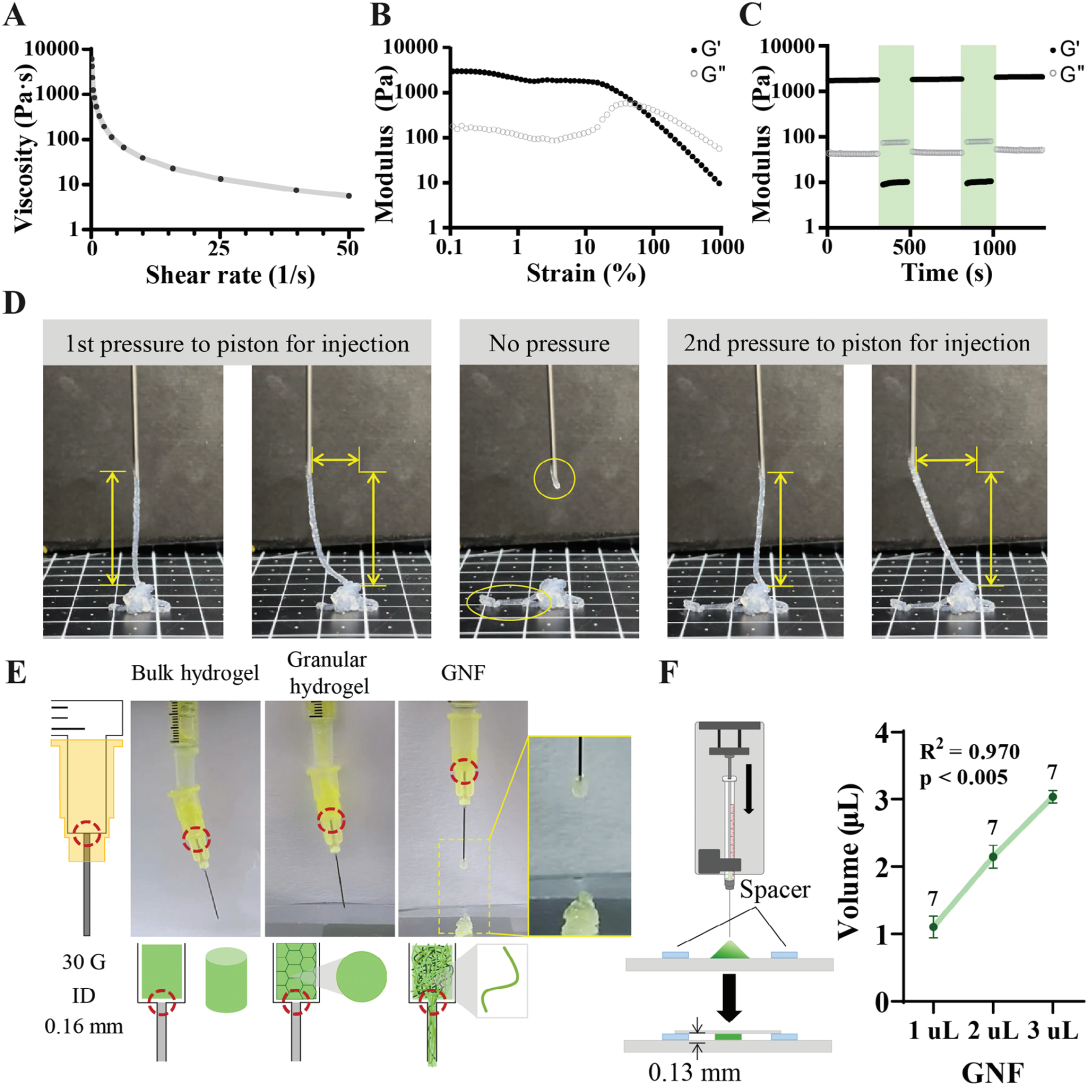
Rheological characteristics and precise volume control of the GNF hydrogel. Rheological characteristics of GNFs showing A) decreasing viscosity with increasing shear rate (0–50 1/s), B) shear‐yielding with increasing strain (0.037–1000 %, 1 Hz), and C) shear‐thinning and self‐healing during low‐strain (1 %, unshaded regions) and high‐strain (500 %, shaded regions) cycles at 1 Hz. D) Images captured during injection, needle translation to the left, and breakage of the GNF hydrogel with/without pressure. The yellow arrows indicate the height and width of the injected strand under pressure, and the yellow circles indicate the breaking and stacking of the strands when the pressure on the piston is removed. Each box in the grid has edges of 1 cm. E) Photographs showing the injectability of the GNF hydrogel as the sub‐microscale GNFs are smaller than the ID of the 30 G needle (0.16 mm). In contrast, bulk hydrogel and granular hydrogels samples cannot be injected because of their larger components. The red circles indicate the joints between the syringe and needle, through which the hydrogels need to pass during injection. The yellow box indicates the moment of injection and stacking of the GNF hydrogel. F) Schematic of the method used to precisely inject 1 µL doses of the GNF hydrogel (*n* = 7, *p* < 0.005) using a syringe pump at 1 µL min^−1^ with a 0.13 mm spacer. The numbers above each dot represent the sample size (*n*). All data are presented as the mean ± SEM.

To confirm that the jammed GNF hydrogel was injectable, its rheological properties were assessed. The jammed GNF hydrogel exhibited shear‐thinning properties, i.e., the viscosity decreased with increasing shear rate (**Figure** **3**A). The hydrogel was elastic at low‐to‐moderate strains and became viscous at high strains (Figure 3B). In addition, it exhibited rapid and reversible transitions in elasticity and viscosity when cycled between high and low strain (see the time points before 500 and 1000 s in Figure 3C). These analyses indicate that the jammed GNF hydrogel could flow during the application of force and recover its viscosity upon force removal. GNF successfully flowed through an 18 G needle under pressure and maintained its strand shape even after leaving the needle, demonstrating its superior injectability (Figure 3D). The GNF strands were maintained with a vertical extension (strand extension to ≈3 cm) and lateral movement of the needle (≈1.3 cm) under pressure and separated from the needle when the pressure was removed. A force exceeding the interactions among the jammed fibers at a weak interaction point broke the strands of the jammed hydrogel. The injectability of the jammed GNF hydrogel was compared to those of GelNB hydrogels of other shapes, such as bulk hydrogels and jammed granular hydrogels (Figure 3E; Movie S1, Supporting Information). Neither the bulk hydrogel nor jammed granular hydrogels passed through a 30 G needle (inner diameter (ID): 0.16 mm) under pressure because of their bulkiness. In contrast, the jammed GNF hydrogel passed through the needle under pressure, and the injected fibers were deposited vertically to the bottom surface.

In addition, the injected volumes of the jammed GNF hydrogel were precisely controlled when injected using a syringe pump (Figure 3F). The jammed GNF was loaded onto glass slides with spacers at each longitudinal edge. The spacers fixed the thickness of the injected samples when the cover glass was gently pushed onto them. The injected volumes were similar to the intended nominal volumes (1 µL: 1.10 ± 0.16 µL, *n* = 7; 2 µL: 2.15 ± 0.17 µL, *n* = 7; 3 µL: 3.04 ± 0.01 µL, n = 7). These results demonstrate that jammed GNF hydrogel can be injected using a syringe at the intended volume, which prevents unwanted tissue damage.

### 2.3 Biodegradability of GelNB Hydrogels

As injectable materials, the degradability of jammed GNFs must be considered. To evaluate the degradability of the jammed GNF hydrogel, we prepared GelNB hydrogel samples (with the same composition as the GNF hydrogel, but in a non‐fibrous form) in a cylindrical shape with a diameter of 8 mm and thickness of 2 mm, incubated them in PBS or PBS containing 1.25 U mL^−1^ collagenase, and freeze‐dried them. These hydrogels were immersed in 1 mL of PBS or PBS with collagenase at 37 °C, and their degradation was monitored over 3 days. The hydrogels in PBS exhibited minimal degradation, with dimensions similar to those of the hydrogels before degradation (Figure S1A, Supporting Information). In contrast, the hydrogels in PBS with collagenase exhibited a significant thickness reduction, eventually forming a powder after centrifugation and freeze‐drying for 3 days, indicating extensive degradation. Quantitatively, the degradation extent was ≈7.74 % for the PBS group and 72.43 % for the PBS + collagenase group (PBS 0 day: 0.00 ± 0.00 %, n = 5; PBS 1 day: 2.39 ± 0.95 %, n = 5; PBS 2 days: 4.92 ± 0.95 %, n = 5; PBS 3 days: 7.74 ± 1.23 %, n = 5; PBS + collagenase 0 day: 0.00 ± 0.00 %, n = 5; PBS + collagenase 1 day: 17.02 ± 1.60 %, n = 5; PBS + collagenase 2 days: 35.58 ± 2.49 %, n = 5; PBS + collagenase 3 days: 72.43 ± 1.58%, n = 5; two‐way repeated measures ANOVA; Figure S1B, Supporting Information). These results suggest that GelNB hydrogels are biodegradable, although the concentration of collagenase used in this experiment was higher than that within the native brain environment. The loaded GFs can be released through both the hydrogel network and as the GNF degrades.

### 2.4 Effects of GNF/GF on IS

**Figure 4:**
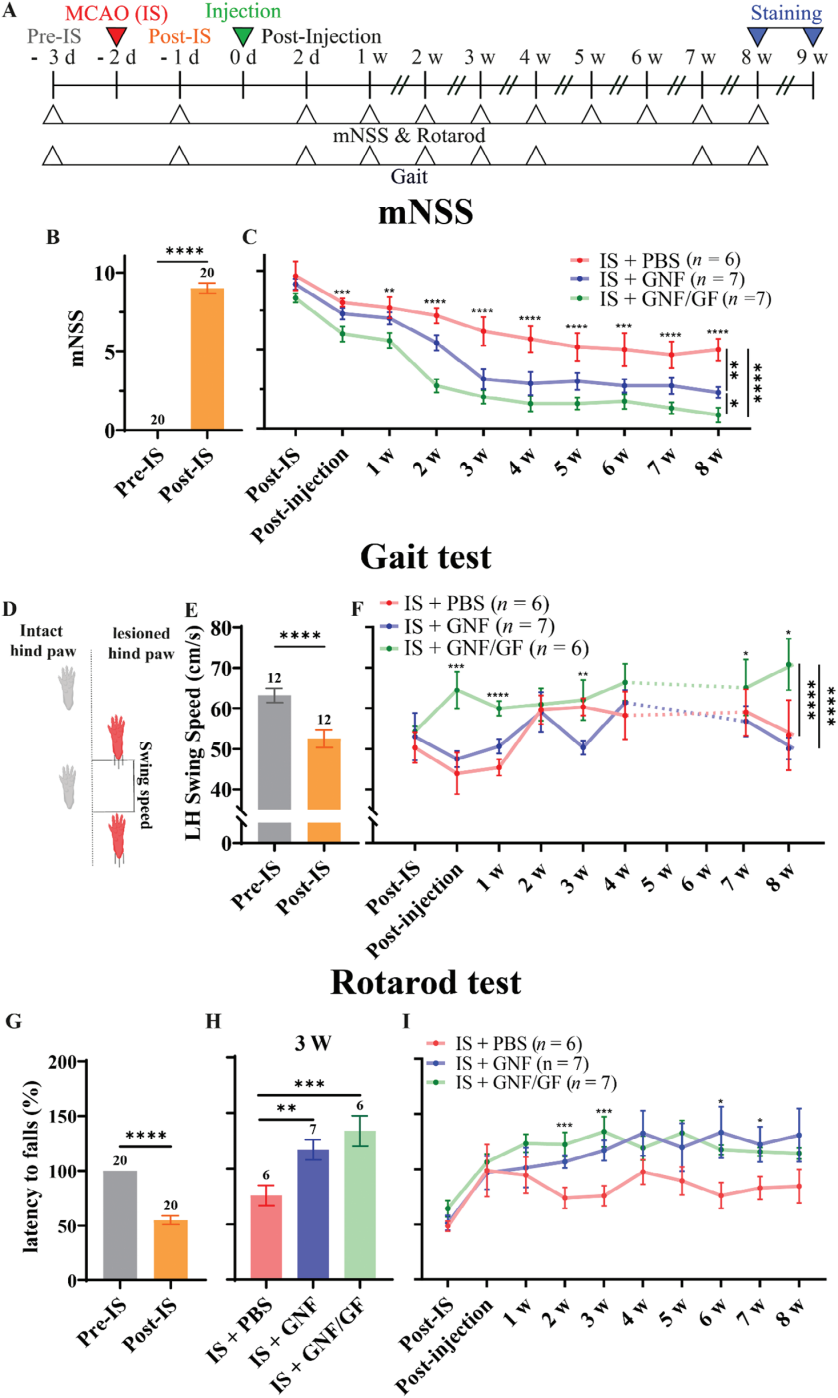
Sensorimotor function alleviation after injection of GNF/GF hydrogel. A) Overall timeline of behavior evaluations. To examine the neurotherapeutic effect of the injectable, jammed GNF/GF hydrogel on IS, we injected PBS, GNF, or GNF/GF into the motor cortex of an IS animal model, which was followed by behavior tests such as mNSS, gait test, and rotarod test for 8 weeks. mNSS tests implemented for the IS models B) pre‐IS and post‐IS (*n* = 20, *p* < 0.005) and C) post‐IS and post‐injection, measured every week for 8 weeks. The IS + GNF (*n* = 7) and IS + GNF/GF (*n* = 7) groups showed a reduced mNSS compared to the IS + PBS group (*n* = 6). D) Schematic describing the swing speeds of intact and lesioned hind paws, which were determined by the duration of each step (time between the paw leaving the ground and then landing). Measured LH swing speeds of the IS models showed E) a significant decrease in the Post‐IS group compared to the Pre‐IS group (*n* = 12, p < 0.005) and F) a significantly faster speed for the IS + GNF/GF group (*n* = 6) compared to the IS + PBS (*n* = 6) and IS + GNF (*n* = 7) groups over the 8 weeks post‐injection. The latency to falls determined by the rotarod test of the IS models showed a G) significantly lower value for the Post‐IS group compared to the Pre‐IS group (*n* = 20, *p* < 0.005). H) IS + GNF (*n* = 7) and IS + GNF/GF groups (*n* = 6) showed significantly higher latency compared to the IS + PBS group (*n* = 6) at week 3 (p = 0.005). I) Latency to falls measured at Post‐IS and Post‐injection states and weekly for 8 weeks after MCAO induction (IS + PBS: *n* = 6; IS + GNF: *n* = 7; IS + GNF/GF: *n* = 6). The number above each bar represents the sample size (*n*) for each group. The *p* values of the behaviors over time were determined by two‐way repeated measures ANOVA with a Bonferroni's post‐hoc test, one‐way ANOVA with a Bonferroni's post‐hoc test for the three groups (weekly tests), and a two‐tailed paired t‐test between Pre‐IS and Post‐IS groups. All data are presented as the mean ± SEM. *p< 0.1, **p< 0.05, ***p< 0.01, ****p< 0.005.

To examine the neurotherapeutic effect of the injectable jammed GNF/GF on IS, it was injected into the motor cortex of an IS animal model, which was followed by behavioral tests such as the modified neurological severity score (mNSS, Table S1, Supporting Information), gait, and rotarod tests (see **Figure** **4**A for the experimental scheme). The mNSS was assessed to test sensorimotor function in IS rats. No significant difference was observed among the PBS, GNF, and GNF/GF groups, with the control rats as a positive control, implying that the injection of GNFs did not influence the mNSS (see Figure S2 and Table S2A, Supporting Information for the statistical details). The mNSS of the IS rats showed a significant increase 1 day after MCAO (Pre‐IS: 0.00 ± 0.00, n = 20; Post‐IS: 9.00 ± 0.32, n = 20; two‐tailed paired sample t‐test; Figure 4B). IS rats treated with GNFs and GNF/GF showed a significant decrease in mNSS compared with the IS + PBS group, with the IS + GNF/GF group exhibiting a lower score than that of the IS + GNF group (Figure 4C; refer to Table S2B, Supporting Information for statistical details). Specifically, the values related to the motor and sensory functions within the mNSS evaluation were higher for the IS + PBS group than those of the IS + GNF and IS + GNF/GF groups, while the balance and reflex parameters of these groups were not significantly different from each other at 8 weeks after injection (Figure S3 and Table S2C, Supporting Information for the statistical details). Additionally, gait was analyzed to evaluate the therapeutic effect of GNF/GF on motor function. The swing speed of the lesioned hind paw (LH) and the time elapsed between the foot leaving the ground and landing were analyzed (Figure 4D). Compared to the Pre‐IS group, the Post‐IS group showed a slower swing speed of the LH (Pre‐IS: 63.19 ± 1.81 cm s^−1^, *n* = 12; Post‐IS: 52.52 ± 2.16 cm s^−1^, *n* = 12; two‐tailed paired sample t‐test; Figure 4E). For 8 weeks, the LH swing speed of the IS + GNF/GF group was significantly faster than that of the IS + PBS and IS + GNF groups (Figure 4F; refer to Table S2D, Supporting Information for statistical details). The latency to falls (determined by the rotarod test) of the IS rats significantly decreased 1 day after MCAO (Pre‐IS: 100.00 ± 0.00, *n* = 20; Post‐IS: 55.08 ± 3.92, *n* = 20; two‐tailed paired sample t‐test; Figure 4G). The IS + GNF and IS + GNF/GF groups showed significantly higher values at week 3 compared to the IS + PBS group (Figure 4H; Table S3A, Supporting Information) and exhibited an upward trend for 8 weeks (Figure 4I; Table S3A, Supporting Information). These results indicate that the injection of jammed GNF and/or GNF/GF hydrogels into the motor cortex of the IS animal model significantly improved the sensorimotor functions.

### 2.5 Histological Effects of GNF/GF

**Figure 5:**
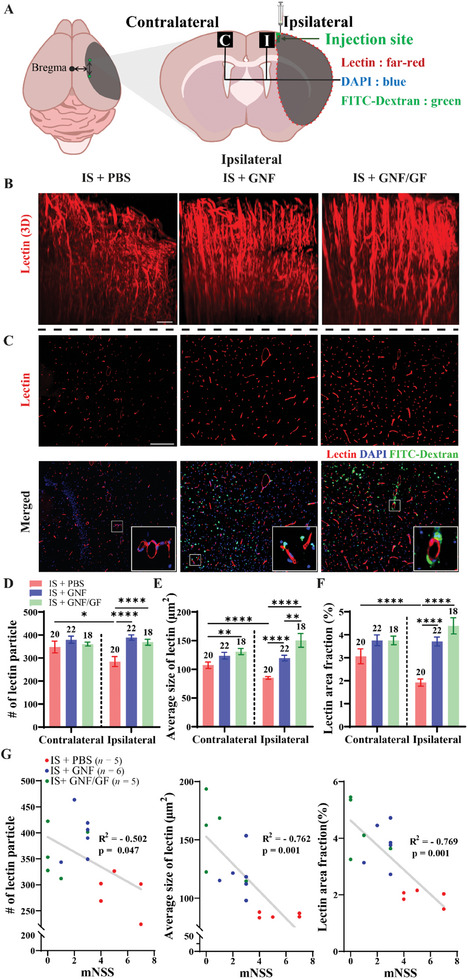
Angiogenesis induced by the GNF/GF hydrogel. A) Schematic of the brains of the IS models showing the injection point (green dots, AP ± 1 mm, ML ‐1 mm, DV +1 mm), analysis area (black boxes, C: contralateral, I: ipsilateral), staining dyes (Lectin: blood vessels; DAPI: nucleus; FITC‐Dextran: GNFs) and infarction region (gray shaded area). B) Lectin (far‐red) labeling of perfusable blood vessels was visualized using fluorescence 3D images of the IS models 8 weeks after injection with PBS, GNF, or GNF/GF. Scale bar: 200 µm. C) DAPI (blue), lectin (far‐red), and GNFs (FITC‐dextran: green) were visualized with fluorescence 2D images of sliced brains obtained 8 weeks after the injection. Representative images of lectin alone (top row) and the merged signals of lectin, DAPI, and FITC‐dextran (bottom row) in the ipsilateral region 8 weeks after injection of PBS, GNF, and GNF/GF groups. Scale bar: 200 µm. D) Number of lectic particles, E) average size of lectin areas, and F) lectin area fraction measured in the contralateral and ipsilateral hemisphere of the IS + PBS (*n* = 20), IS + GNF (*n* = 22), and IS + GNF/GF (*n* = 18) groups. G) Scatter plots showing the inverse correlations between mNSS and the number of lectin particle (left), average size of lectin areas (middle), and lectin area fraction (right). Each data point represents an individual animal. The number above each bar represents the sample size (*n*) for each group. The p values were determined by one‐way ANOVA with a Bonferroni's post‐hoc test for the three ipsilateral groups, and two‐tailed independent samples t‐test were used to compare between contralateral and ipsilateral hemisphere data from the same group. All data are presented as the mean ± SEM. *p< 0.1, **p< 0.05, ***p< 0.01, ****p< 0.005.

To investigate if the jammed GNF/GF induces angiogenesis in the brain of the IS model, fluorescent images of perfusable blood vessels were analyzed in contralateral and ipsilateral regions (marked by the black boxes in **Figure** **5**A; Anteroposterior (AP): ± 1 mm, Mediolateral (ML): −1 mm, Dorsoventral (DV): +1 mm) of IS + PBS, IS + GNF, and IS + GNF/GF groups. Lectin (far‐red) labeling of perfusable blood vessels was visualized using fluorescence 3D images to show the overall distribution of blood vessels (Figure 5B), and the lectin content was quantified using fluorescence 2D images. The microvessel structure in the 3D images was less collapsed for the IS + GNF and IS + GNF/GF groups than for the IS + PBS group. In addition, 4',6‐diamidino‐2‐phenylindole (DAPI: blue), lectin (far‐red)‐labeled perfusable blood vessels, and GNFs (fluorescein isothiocyanate (FITC)‐dextran: green) were visualized in fluorescence 2D images of sliced brains obtained 8 weeks after injection (Figure 5C). The injection of GNF and GNF/GF hydrogels increased the number of lectin particles, defined as a lectin area larger than 30 µm^2^ (Figure 5D; refer to Table S3B, Supporting Information for statistical detail), the average size of the lectin particles (Figure 5E; refer to Table S3C, Supporting Information), and lectin area fraction (Figure 5F; refer to Table S3D, Supporting Information) at ipsilateral regions. This indicates that either the GNFs or GFs contributed to the formation of blood vessels. Interestingly, the GNF/GF group showed a higher average lectin size compared to the GNF group. From the images of the ipsilateral regions, decreasing the mNSS correlated with a increase in the number of lectin particles, average size of lectin, and lectin area fraction (Figure 5G). This indicates that the recovery of sensorimotor function is correlated with an increase in angiogenesis. Additionally, ionized calcium‐binding adapter molecule 1 (Iba‐1) protein, a microglial marker, and glial fibrillary acidic protein (GFAP), an astrocyte marker, were used to observe the effect of GNFs on inflammation. The IS + PBS and IS + GNF groups showed a significant increase in microglia and astrocytes on the ipsilateral side compared to the IS + GNF/GF group (Figure S4 and Table S3E, Supporting Information), demonstrating that the latter decreased inflammation. Infarct volumes were also analyzed to test whether the administration of GNF/GF affected the disruption of the brain of IS rats 2 days after MCAO and 8 weeks after PBS, GNF, and GNF/GF injections (Figure S5A, Supporting Information). No significant differences in the infarct volumes were observed among the groups (IS (2 d): 39.4249 ± 7.19 %; IS + PBS: 48.86 ± 4.00 %, *n* = 5; IS + GNF: 42.00 ± 0.58 %, *n* = 5; IS + GNF/GF: 43.49 ± 3.37 %, *n* = 5; one‐way ANOVA with Bonferroni's post‐hoc test; Figure S5B, Supporting Information).

## 3 Discussion

In this study, a jammed GNF hydrogel, possibly with small pores between the GNFs, was shown to be injectable with precise volume control, and improved angiogenesis and sensorimotor functions in a IS animal model. The jammed GNF hydrogel meets the current demands for treatment in the brain such as minimal invasiveness, scalability, and target specificity because the nanoscale diameters of the GNFs permit the injection of ≈1 µL through 30 G needles (ID: 0.16 mm). According to previous studies,^[^ ^25^, ^26^ ^]^ the Young's modulus of the jammed GNFs is comparable to that of brain tissue. Additionally, the force used to inject the jammed GNFs is similar to that used to inject the hydrogel precursor in a previous study.^[^ ^26^ ^]^ These characteristics enable the injection of neurotherapeutic drugs deep into brain areas that are difficult to access surgically. In addition, the jammed GNF hydrogel is mainly composed of GelNB, making it biocompatible and suitable for pharmacotherapeutics.^[^ ^27^, ^28^ ^]^ To date, no negative effects of injectable GNFs on the brain have been observed.

In this study, the injection of GNFs alone induced angiogenesis and improved sensorimotor function (as measured by the mNSS). GNF/GF treatment was more effective than GNF alone (as measured by gait and lectin analyses). Angiogenesis is not solely induced by empty hydrogels.^[^ ^12^, ^13^, ^14^ ^]^ Our results imply that the pure GNF hydrogel acts as an ECM‐like fibrous network for growth of endothelial cells, which might be useful for tissue regeneration via the interaction between the GNFs and endothelial cells in the lesioned area, thereby promoting angiogenesis and increasing sensorimotor functions. Additionally, gelatin, the main component of GNFs, appears to be helpful in tissue regeneration; peripheral nerve,^[^ ^29^, ^30^ ^]^ tendon,^[^ ^31^ ^]^ and skin^[^ ^32^ ^]^ tissues were regenerated with gelatin hydrogels (which were not carrying GFs or drugs).

We demonstrated that angiogenic factors released from the GNF hydrogel augmented the average size of lectin‐positive blood vessels in the brain and improved sensorimotor functions, including the LH swing speed. In previous studies, VEGF, PMA, and S1P were found to be major players in angiogenesis.^[^ ^33^, ^34^, ^35^, ^36^, ^37^, ^38^ ^]^ Elevated levels of VEGF contribute to an increase in blood vessel diameter^[^ ^38^ ^]^ and promote the formation of blood vessels from pre‐existing vessels by promoting endothelial cell migration and differentiation.^[^ ^39^ ^]^ VEGF also maintains and restores nerves in an ischemia state,^[^ ^40^ ^]^ leading to improvements in motor function.^[^ ^41^ ^]^ S1P signaling plays an important role in maintaining the function of microvessels during IS^[^ ^34^ ^]^ and regulates cell migration and sprout multicellularity.^[^ ^42^ ^]^ PMA promotes pro‐angiogenic signaling by activating protein kinase C, which subsequently activates matrix metalloproteinases‐2 and ‐9.^[^ ^43^ ^]^

The enhanced anti‐inflammatory effect of the IS + GNF/GF group is probably due to the effects of S1P, VEGF, and angiogenesis as evidenced by the larger average size of lectin‐positive blood vessels in this group compared to other groups. VEGF is responsible for immunosuppression by inducing programmed death‐ligand 1 expression^[^ ^39^ ^]^ and S1P inhibits vascular inflammation.^[^ ^44^ ^]^ In a previous study, IS led to a hypoxic state, which induced inflammation and gradually increased the number of microglia and astrocytes over 1 month.^[^ ^45^ ^]^ In the IS + PBS and IS + GNF groups, higher microglia and astrocyte counts were observed in the lesioned hemispheres compared to the GNF/GF group. Therefore, the enhanced angiogenesis by the GFs could rapidly alleviate the hypoxic state, reducing the degree of inflammation. These effects synergistically induce robust endothelial cell sprouting and new blood vessel formation^[^ ^33^ ^]^, rapidly alleviating the hypoxic state, reducing the degree of inflammation, and enhancing sensorimotor function.

## 4 Experimental Section

### Synthesis of Gelatin‐Norbornene

GelNB was synthesized according to a previous method.^[^ ^28^ ^]^ Briefly, exo‐5‐norbornene carboxylic acid, N, N’‐dicyclohexylcarbodiimide and N‐hydroxysuccinimide were transferred to anhydrous N, N‐dimethylforamide via cannulation in final concentrations of 1.215, 1.013, and 1.519 mmol, respectively, and incubated at 60 °C for 24 h to obtain the activated form of exo‐5‐norbornene succinimidyl ester. Then, 150 mL of anhydrous dimethyl sulfoxide (DMSO) was transferred to 5 g of gelatin type A, and the gelatin was fully dissolved at 40 °C. The gelatin dissolved in DMSO was mixed with the activated form of exo‐5‐norbornene succinimidyl ester at 60 °C for 24 h. Precipitates obtained by precipitating the mixture in acetone and filtering were fully dissolved in deionized water (DW) at 60 °C. The aqueous mixture was dialyzed for at least 72 h, followed by pH adjustment to 7.4. Finally, the mixture was frozen at ‐80 °C overnight and lyophilized for at least 3 days.

### TNBSA Assay

Gelatin and GelNB were dissolved in reaction buffer (0.1 M sodium bicarbonate, pH 8.5) at concentrations of 1, 0.75, 0.5, 0.25, 0.1, and 0 mg mL^−1^. Then, 0.25 mL of 0.01% (w/v) TNBSA was added to 0.5 mL of gelatin and GelNB in the reaction buffer. All samples were incubated in a water bath set to 37 °C for 2 h. Then, 0.25 mL of 10 % sodium dodecyl sulfate and 0.125 mL of 1 N HCl were added to the samples, and their absorbance at 335 nm was measured. Absorbance at each concentration was calculated to make standard curve of sample. The following formula was used to calculate the degree of substitution (DS):

(1) $$DS\;\left(\%\right)=\left(1-\frac{slope\;of\;synthesized\;material}{slope\;of\;gelatin}\right)\times 100$$

### NMR Analysis

^1^H NMR analysis was performed using a Bruker TopSpin4.3.0 system. Gelatin was used as a calibration standard at room temperature (RT). Gelatin and GelNB samples were dissolved in D_2_O at a concentration of 10 % (w/v) and then transferred to NMR tubes with 5 mm diameters. All spectra were recorded using an NMR Bruker AVANCE 400 MHz III HD. The DS was then analyzed by comparing the norbornene double‐bond proton signals at 6.28 and 6.171 ppm and the methyl proton (Val, Leu, and Ile) signals at 1.01 ppm.

### Fabrication of Electrospun Fibrous Hydrogel Mats

First, ≈2 mL of 2.5 wt.% PEO dissolved in DW was electrospun using a 1 mL syringe with an 18 G metal needle. For the electrospinning of the PEO precursors, the rotation speed of the grounded collector covered with aluminum foil, distance from the needle tip to the collector, flow rate, and voltage were 100 rotations per minute (rpm), 19 cm, 1.2 mL h^−1^, and 11 kV, respectively. Then, 7 wt.% GelNB, 2.5 wt.% PEO (900 kDa), 0.1% (v/v) Irgacure 2959, 0.05% (v/v) FITC‐Dextran (MW 2,000,000), and 0.65 wt.% 4‐arm PEG‐thiol were dissolved in DW at 35 °C for 4 h and loaded into a 1 mL syringe with a 18 G metal needle. The GelNB and PEO precursors were simultaneously electrospun. The distance from the needle tip to the collector, flow rate, and voltage were 20 cm, 0.8 mL h^−1^, and 20 kV for GelNB precursor electrospinning, respectively, and the conditions for the PEO precursor electrospinning were the same as those described earlier. The rotation speed of the collector, temperature and humidity were 100 rpm, 40 °C, and 10 %, respectively. Each side of the GelNB/PEO fiber mats cut into 2 × 2 cm pieces was exposed to UV light (320–390 nm; OmniCure S1500 UV) for 1 h. Instead of GelNB precursors, GelNB precursors containing VEGF, S1P, and PMA at concentrations of 200 ng mL^−1^, 1000 nM and 1200 ng mL^−1^, respectively, were used to fabricate the electrospun fibers containing GFs.

### Fabrication of Injectable GNF Hydrogels

The electrospun mats were hydrated in 1 mL of PBS to remove the PEO fibers. The solutions with GNFs were repeatedly extruded from 18, 22, and 26 G needles until the extrusion through the needles became smooth. Jammed GNFs were obtained by centrifuging the solutions at 17,500 rpm for 2 min and removing the supernatant.

To analyze the fragmented GNFs, 10 µL of GNF solution from the 18, 22, and 26 G needles was diluted in 100 µL of DW. Then, 2 µL of diluted GNF solution was dropped onto a slide glass and covered by a coverslip. Images were captured using an inverted microscope (IX71; Olympus, Japan). The diameter of the GNFs was measured by ImageJ software based on the full width at half maximum of ten random fibers from one of the fluorescent images X 5 (total of fifty fibers from five images at each G (n = 50).

To demonstrate the injectability of the GNF hydrogel, a GelNB bulk hydrogel, jammed granular hydrogels, and GNFs were injected through a 30 G needle. Specifically, the GelNB precursor was loaded into a 1 mL syringe with a 30 G needle and polymerized via 3 min of UV light exposure. A syringe loaded with GelNB bulk hydrogel was mounted into a syringe pump and pumped at a flow rate of 1 µL min^−1^. GelNB precursor on a Petri dish was polymerized by 3 min of UV light exposure, and the GelNB hydrogel was ground using a spatula to form granular hydrogels. The GelNB granular hydrogels jammed by vacuum filtration was injected using the aforementioned method. Similarly, GNFs were injected using the aforementioned methods. All injection times were recorded using a camera (iPhone SE2) and the granular hydrogels size was obtained by imaging with an Olympus inverted microscope.

To analyze the injection volume, the GNFs loaded in a Hamilton syringe with a 30 G needle were injected between spacers (thickness was 0.13 mm) and onto glass slides with a syringe pump at a flow rate of 1 µL min^−1^. Cover slides were placed on top of the spacers and injected with GNFs, and their edges were gently pushed together. The images of the GNFs were vertically captured by a camera. The areas of the GNFs were analyzed using ImageJ. The injected volumes were calculated by multiplying the area by the spacer thickness. Injections of 1, 2, and 3 µL of GNFs were repeated 7 times and the injection volumes were statistically analyzed using GraphPad Prism.

### Rheological Characterization

A rheometer (TA, DHR‐20) was used to measure the rheological properties of the jammed GNFs. A jammed GNF sample was placed on a Peltier plate and covered by a 20 mm parallel plate with a 1 mm gap. The measurements were performed at room temperature. The viscosity was measured as the shear rate increased from 0 to 50 1/s and the modulus was measured under strains of 0.037–1000 % at 1 Hz to analyze the shear‐thinning properties. By applying periodic strain changes between 1 % and 500 % at 1 Hz, shear‐thinning and self‐healing properties were observed.

### In Vitro Degradation Tests

The enzymatic degradation method was used to study the in vitro degradation rate of the bulk GelNB hydrogels. A GelNB precursor containing 7 wt.% GelNB, 0.65 wt.% 4‐arm PEG‐thiol, and 0.1%(v/v) Irgacure 2959 was poured into a Petri dish and polymerized for 10 min under UV light exposure. GelNB hydrogel samples were prepared using an 8‐mm biopsy punch and freeze‐dried for 1 day. The freeze‐dried hydrogels were suspended in PBS (Ctrl) or PBS containing 1.25 U mL^−1^ collagenase (Type IV, ≥ 125 collagen digestion units (CDU)/mg solid) after measuring their initial weight (*W_1_*). The samples were subsequently placed in an oven at 37 °C in the dark. Every day, each sample was freeze‐dried for 1 day, and the weight (*W_2_*) was measured. The experiments were conducted five times. The degradation percentage was calculated using the following equation:

(2) $$Degradation\%=\left(\frac{W_{1}-W_{2}}{W_{1}}\right)\times 100$$

### Animals

All animal procedures were approved by the Institutional Animal Care and Use Committee of the Incheon National University (INU‐ANIM‐2017‐08). Male 10 weeks old Sprague–Dawley (SD) rats (280‐300 g) were used in these experiments. The SD rats were housed under a 12 h dark/night cycle and provided free access to water and food.

### MCAO‐Induced Rats

The SD rats were anesthetized with 5 % isoflurane in a mixture of 70 % N_2_ and 30 % O_2_ for 3 min. The concentration of isoflurane was decreased to 2–3 % during surgery. To induce MCAO in the SD rat brains, the right external carotid artery (ECA), common carotid artery (CCA), and internal carotid artery (ICA) were exposed. Blood flow in the ECA was permanently restricted using sterilized sutures (black silk, AILEE). Blood flow in the ICA and CCA was temporarily restricted using sterilized sutures. Then, 6 mm silicon rubber‐coated monofilament (4‐0 Medium MCAO suture, L.M.S. KOREA) was inserted into the ICA to a depth of 1.9 cm through the ECA. The monofilament was withdrawn after 90 min. All SD rats received 5 mg kg^−1^ ketoprofen after surgery. The SD rats were maintained at 37 °C with an infrared lamp.

### Injection of PBS, GNF, and GNF/GF

The SD rats were anesthetized with isoflurane. An ophthalmic ointment was applied to the eyes to prevent them from drying out, and the fur on the head was shaved. The heads of the rate were fixed on a stereotaxic apparatus (Soelting, Illinois, USA) with a stereotaxic adaptor and their body temperature was maintained at 37 °C with a temperature‐controlled heating pad during surgery. The head was sterilized thrice with alternating alcohol and povidone scrubs. Then, 0.1 mL of lidocaine (2 %) was injected into the scalp and a 2 cm incision was made. The skull was scratched to remove the periosteum. The skull was drilled to make two holes (AP ± 1 mm, ML ‐1 mm, DV +1 mm). Then, 2 µL of PBS, GNF, or GNF/GF was injected through the holes using a microsyringe pump. After injection, the syringe was raised by 0.5 mm using a stereotaxic arm and left for 5 min. The syringe was removed, and the holes were sealed with bone wax (Bonewax, Ethicon, New Jersey, USA). The incised scalp was adhered using a biocompatible adhesive (Vetbond, 3 M, Minnesota, USA). After surgery, the SD rats received 5 mg kg^−1^ ketoprofen and their body temperature was maintained at 37 °C using an infrared lamp.

### mNSS Tests

The mNSS test was performed to evaluate neurological function according to a previous method.^[^ ^52^ ^]^ The mNSS ranges from 0 to 18, with higher scores indicating more severe conditions. The mNSS consists of motor, sensory, beam balance, and reflex absence tests (Table S1, Supporting Information). The mNSS was implemented when rats were in the pre‐IS, post‐IS, and post‐injection states, and every week for 8 weeks after injection. The pre‐IS and post‐IS states indicate the time points one day before and after MCAO induction, respectively. The post‐injection state refers to 2 days after injection.

### Gait Test

Quantitative gait analysis was performed to evaluate locomotor function. A transparent walkway 100 cm in length and 17 cm wide was used, with a camera positioned below the walkway. The walking task was recorded until satisfactory walking was achieved without pause. During the test, rats were allowed to move freely along the walkway. Rats were subjected to gait assessment in the post‐IS and post‐injection states, and at 1, 2, 3, 4, 7, and 8 weeks after PBS, GNF, or GNF/GF treatments. During gait analysis, the paws were labeled as intact (right side) and lesioned (left side). The swing speed (cm s^−1^) of the LH was measured. All gait analyses were conducted using a Tracker (https://physlets.org/tracker/).

### Rotarod Test

The SD rats underwent a 3day training period on a rotarod (Rotarod for Rats, Ugo Basile) at a speed of 4 revolutions per minute (rpm). The test was conducted in the accelerating mode, in which the rotating speed was increased from 4 to 40 rpm in 300 s. The test was performed three times and the average duration was calculated, with the time measured until the rats fell off the rotarod. Trials in which the rats intentionally jumped off were excluded from the analysis. The first measurement (Pre‐IS) was used as the baseline.

### Lectin Perfusion

The SD rats were anesthetized with 5 % isoflurane. The abdominal wall and the integument beneath the rib cage were incised. A small perforation was made in the arachnoid mater, which was separated from the ribcage. Both sides of the ribcage were incised, removed, and secured using a needle to expose the pleural cavity. *Lycopersicon esculentum* agglutinin (tomato lectin, 75 µg/100 µL, L32472, Invitrogen, Carlsbad, CA, USA) was injected into the left ventricle. After 2 min of incubation, the right atrium was incised to create an outlet. Then, 100 mL of saline was perfused, along with 80 mL of 4 % paraformaldehyde (PFA).

### Paraffin Embedding

The brain was stored in 4 % PFA at 4 °C overnight, the 4 % PFA was changed to 1X PBS, and stored at 4 °C overnight. The brain was sectioned into 3 mm slices using a rat brain matrix (RWD, Guangdong, China). The brain slices were placed in a cassette and the following steps were performed: 70 % ethanol for 15 min, 95 % ethanol for 15 min, 100 % ethanol for 15 min × 2, 100 % ethanol for 30 min, 100 % ethanol for 45 min, xylene for 20 min × 2, xylene for 45 min, paraffin for 30 min × 2 at 60 °C, paraffin for 45 min at 60 °C. The brain slices were secured into the cassette using an embedding station (HistoCore Arcadia H – Heated Paraffin Embedding Station, Leica) and a cold plate (HistoCore Arcadia C – Cold Plate, Leica). The resulting paraffin‐embedded brains were sectioned at 4 µm using an automated rotary microtome (HistoCore Autocut, Leica). The sectioned slices were floated onto 38 °C DW. The slices were mounted on glass slides (Histobond adhesive microscope slides, Marienfeld) and dried overnight.

### Cresyl Violet Staining

For deparaffinization and cresyl violet staining, the following steps were performed: xylene for 2 min × 2, 100 % ethanol for 2 min × 3, 95 % ethanol for 2 min, 70 % ethanol for 1min, 50 % ethanol for 1 min, DW for 2 min, DW for 1 min, 0.1 % cresyl violet at 50 °C for 20 min, DW for 2 min, DW for 1 min, 70 % ethanol for 2 min, 95 % ethanol for 5 min, 95 % ethanol for 2 min. After these steps, a mounting solution (Mount‐Quick, Daido‐Sangyo Co.) was applied to the brain slice, which was then covered with a glass coverslip.

### Infarct Volume Analysis

The infarct volumes were analyzed following a previous method.^[^ ^53^ ^]^ The images from at least four brain slides were used to calculate the infarct volumes using ImageJ 1.47v software (National Institutes of Health, Bethesda, MD, USA; https://imagej.nih.gov/ij/; accessed on 20 March 2021) and the following formula:

(3) $$\%I_{m}=\frac{\sum \left\{\;\left(V_{c}\;-\;V_{I}\right)/\;V_{c}\right\}\;}{n}\times 100$$

where %*I*_m_ is the average infarct volume percentage, *V*_c_ and *V*_I_ are the volumes of the contralateral and ipsilateral hemispheres, respectively, and *n* is the number of brain slices.

### Immunohistochemistry

Deparaffinization of the brain slices was performed. The brain slices were incubated with 0.01 mol L^−1^ citrate buffer for 30 min at 80 °C and washed in 0.3 % triton‐X buffer for 15 min at RT. Then, the brain tissues were incubated with primary antibodies against Iba‐1 (1:1000, 019‐19741, Wako, Osaka, Japan) or GFAP (1:1000, G6171, Sigma, Massachusetts, USA) overnight at 4 °C. After 18 h, the brain tissues were washed in 1X PBS buffer for 30 min and incubated with the following secondary antibodies at RT for 2 h: goat anti‐rabbit Alexa Fluor 594 (1:1000, A11012, Invitrogen, Carlsbad, CA, USA) or anti‐mouse Alexa Fluor 594 (1:1000, A‐11005, Invitrogen, Carlsbad, CA, USA) and were washed in 1X PBS buffer for 30 min. The sections were then placed on glass slides and mounted with Fluoroshield Mounting Medium containing DAPI (ab104139, Abcam, Cambridge, UK) and covered with a coverslip to prevent the movement and drying of the samples.

### Image Capture and Analysis

Images were acquired using a fluorescence microscope (Axioplan2 Imaging; Carl Zeiss Microimaging Inc., Oberkochen, Germany). For immunohistochemistry analyses, the sections were anatomically matched to the rat brain atlas. Both sides of the bilateral brain regions were analyzed in four brain sections (two slices per injection site) per rat. Using ImageJ 1.47v software, an unbiased observer counted the number of Iba‐1‐positive microglial cells and GFAP‐positive astrocytes and analyzed the number of lectin particles, average size of lectin particles, and lectin area fraction.

### Clarity and 3D Imaging

The 4 % PFA‐fixed MCAO rat brains were cleared using the rapid tissue‐clearing method (Binaree Inc., Daegu, Republic of Korea) following the manufacturer's instructions. Briefly, the fixed rat brains were cleared in an electrophoresis chamber (Binaree Tissue‐Clearing Rapid Chamber) for 4 h. Next, the cleared rat brains were transferred to an tissue clearing solution for imaging (termed AICI) mounting solution^[^ ^54^ ^]^ and agitated at 50 rpm for 24–36 h at 37 °C. Subsequently, the reflex index was matched to a 3D whole‐brain image and imaged using a spinning‐disk‐type confocal microscope (Dragonfly, Andor Technology, Belfast, UK) with a 4× objective lens (Nikon, Plan Apo λ 0.2 NA). The samples were imaged using an inverted microscope in a glass‐bottomed dish. Confocal microscopy images were rendered into 3D images using Imaris software (ver. 9.2.1, Bitplane).

### Statistical Analysis

Statistical analysis were performed using SPSS 28.0 software (IBM Corp., Armonk, NY, USA). Outliers were handled according to quartiles. All data was expressed as mean ± SEM. Two groups were compared using two‐tailed independent sample t‐tests and two‐tailed paired t‐tests. Comparisons between three groups were conducted using one‐way ANOVA with a Bonferroni's post‐hoc test. The behavioral experiments performed every week and the degradation of GelNB percentage measured over time were analyzed using two‐way repeated‐measures ANOVA with a Bonferroni's post‐hoc test. Statistical significance was defined as p < 0.05 (*p< 0.1, **p< 0.05, ***p< 0.01, and ****p< 0.005.). All graphs were constructed using GraphPad Prism 7 software (GraphPad Software Inc., La Jolla, CA, USA). All illustrations were created using Illustrator CC 2019 (Adobe Inc., San Jose, CA, USA) and Biorender.com.

## Conflict of Interest

The authors declare no conflict of interest.

## Author Contributions

S.Y., K.H.S., and J.G.K. conceptualized the experiments and designed the study. D.K. and J.H.C. performed the behavioral tests and animal surgery. J.W.L. prepared the injectable jammed electrospun hydrogel. D.K. and Y.T.K. performed histology. D.K., J.W.L., Y.T.K., J.H.C., and G.E.K. analyzed the data. C.M.H. performed tissue clearing and 3D imaging. All authors contributed to writing the paper. S.Y., K.H.S., and J.G.K. revised the manuscript.

## Supporting information



Supporting Information

Supplemental Movie S1

Supplemental Movie S1

## Data Availability

The data that support the findings of this study are available on request from the corresponding author. The data are not publicly available due to privacy or ethical restrictions.
