# Supplementary material for: Minimally Invasive Syringe‐Injectable Hydrogel with Angiogenic Factors for Ischemic Stroke Treatment
Source: Adv Healthc Mater. 2024 Nov 9;14(6):2403119. doi: 10.1002/adhm.202403119 (PMC11874675; doi:10.1002/adhm.202403119)
Supplement: Supplementary file 1 — Supporting Information [file ADHM-14-0-s002.pdf]

ADVANCED HEALTHCARE MATERIALS

# Supporting Information

for *Adv. Healthcare Mater.*, DOI 10.1002/adhm.202403119

Minimally Invasive Syringe-Injectable Hydrogel with Angiogenic Factors for Ischemic Stroke Treatment

*Donggue Kim, Ji Woo Lee, Yang Tae Kim, Junhyeok Choe, Gaeun Kim, Chang Man Ha, Jae Geun Kim*, Kwang Hoon Song* and Sunggu Yang**

## Supplementary Materials

**Movie S1. Injectability of jammed GNF.** The jammed GNF hydrogel could be injected through a 30 G needle (ID: 0.16 mm), while the bulk hydrogel and jammed granular hydrogels of GelNB could not be injected.

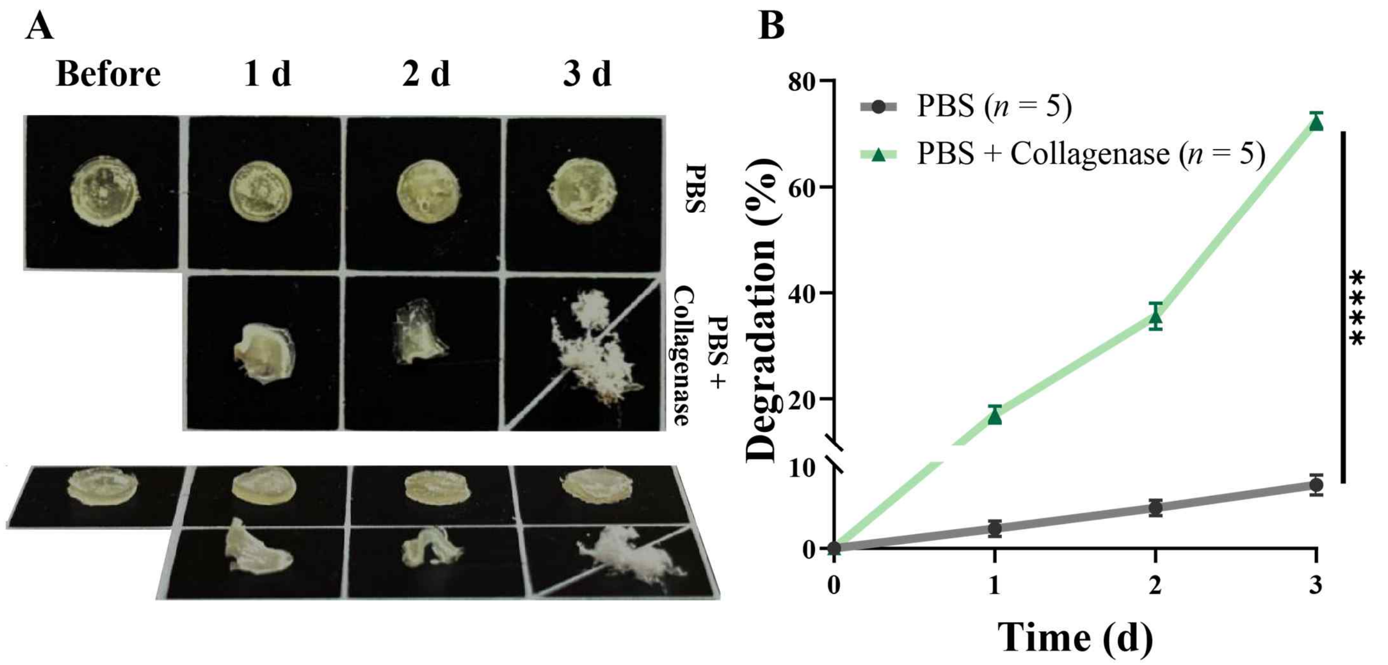


**Fig. S1. Degradation of GelNB hydrogels in PBS and PBS containing 1.25 U/mL collagenase. (A)** Representative photograph pictures of degradation and **(B)** degradation percentage of lyophilized GelNB hydrogels incubated in PBS and PBS + collagenase (*n* = 5, $p < 0.005$). The p values of the degradation over time were determined by two-way repeated measures ANOVA tests. All data are presented as the mean ± SEM. *p < 0.1, **p < 0.05, ***p < 0.01, ****p<0.005.

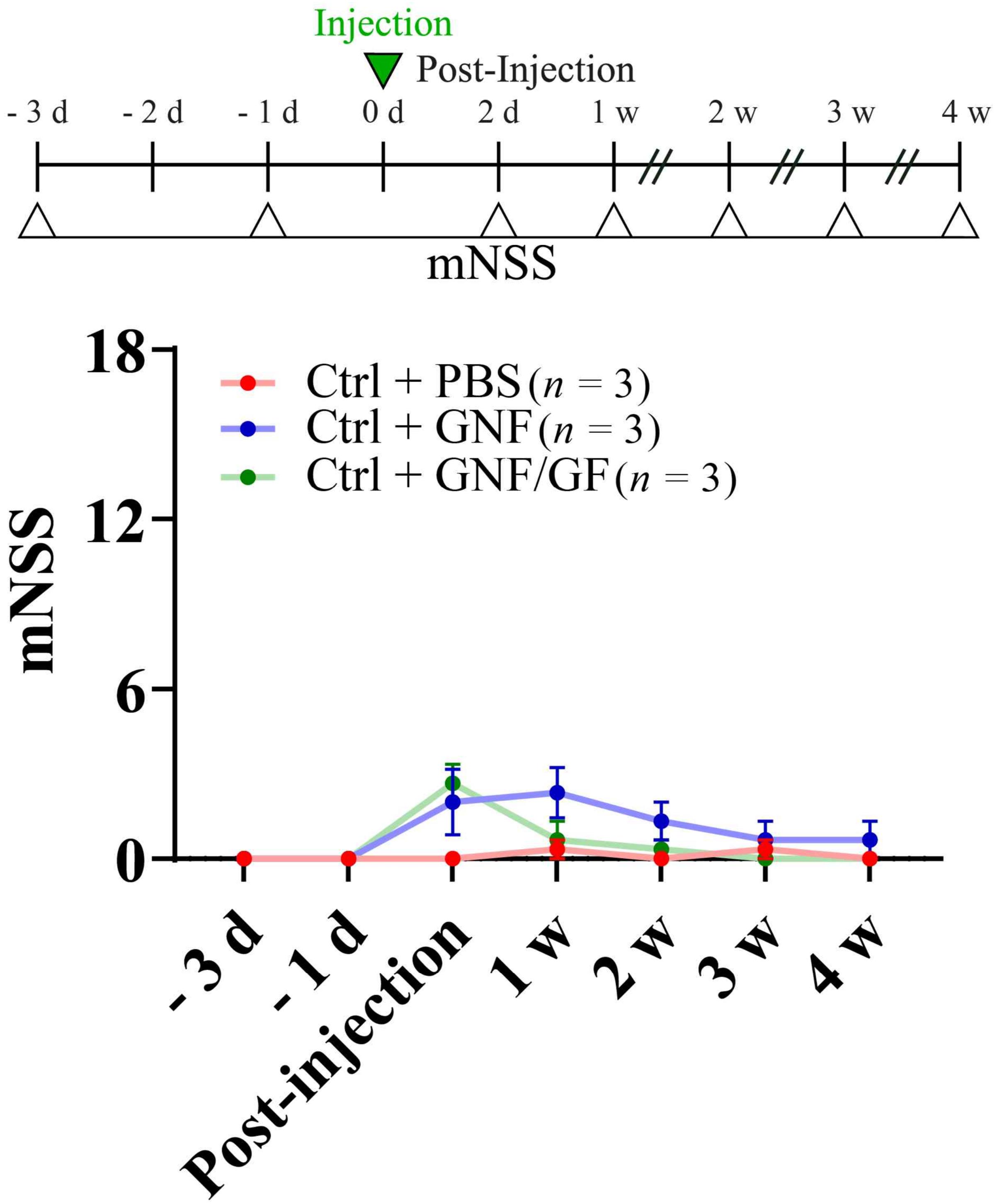


**Fig. S2. Measurement of mNSS after injection of PBS, GNF, or GNF/GF in the control group.** Timeline of measuring the mNSS scores. No significant differences were observed between the groups. The p values were determined by two-way repeated-measures ANOVA with Bonferroni's post-hoc test for the three groups ($n = 3$). All data are presented as the mean ± SEM. *$p < 0.1$, **$p < 0.05$, ***$p < 0.01$, ****$p < 0.005$.

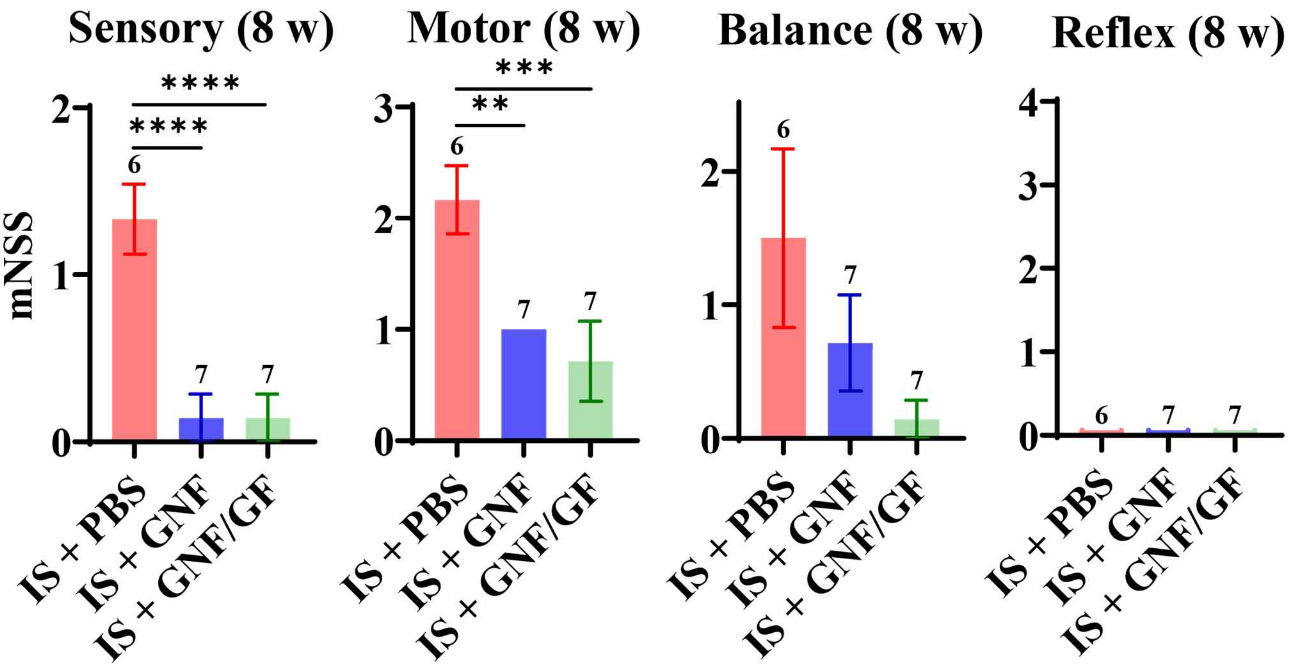


**Fig S3. Individual mNSS parameters measured 8 weeks post-injection for the three** IS **treatment groups.** The sensory and motor function parameters for the IS + PBS group (*n* = 6) are higher than those for the IS + GNF (*n* = 7), and IS + GNF/GF groups (*n* = 7). Numbers above each bar represent the sample size (*n*) for each group. The p values were determined using one-way ANOVA with Bonferroni's post-hoc test between the three groups. All data are presented as the mean ± SEM. *p < 0.1, **p < 0.05, ***p < 0.01, ****p < 0.005.

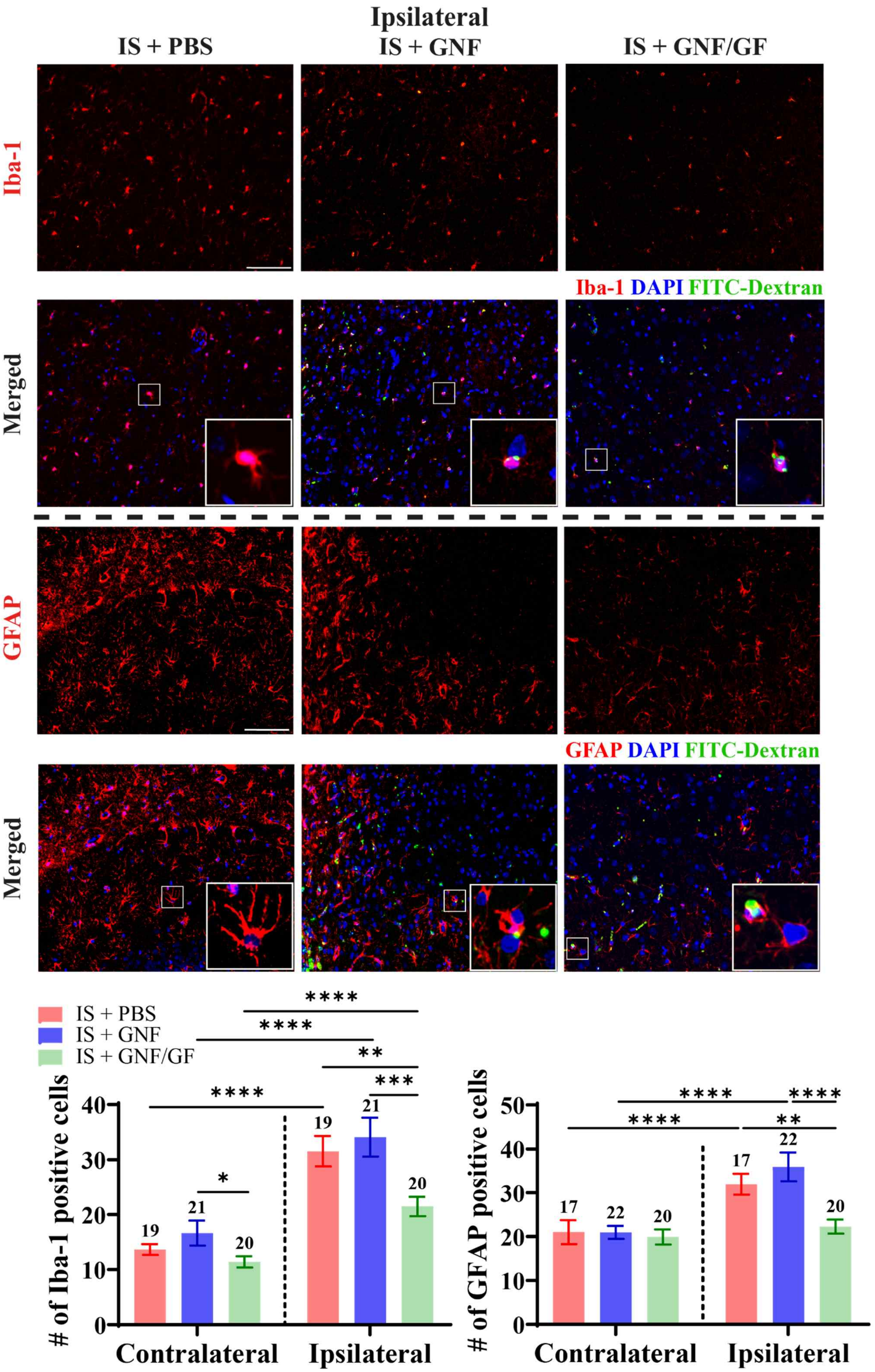

Ipsilateral
IS + PBS
IS + GNF
IS + GNF/GF
Iba-1
Iba-1 DAPI FITC-Dextran
Merged
GFAP
GFAP DAPI FITC-Dextran
Merged
IS + PBS
IS + GNF
IS + GNF/GF
# of Iba-1 positive cells
# of GFAP positive cells
Contralateral
Ipsilateral
Contralateral
Ipsilateral

**Fig. S4. Long-term change of glial cells in the three groups.** Representative images of the ipsilateral hemisphere above the black dashed line, Iba-1 (red), a marker of microglia; FITC-dextran (green), a marker of GNFs; and DAPI (blue), a marker of nuclei. GFAP (red), a microglial marker, FITC-dextran, and DAPI are shown below the black dashed line. The numbers of lba-1 and GFAP positive cells were measured in the contralateral and ipsilateral hemispheres of the three groups. The IS + PBS (*n* = 19) and IS + GNF (*n* = 21) groups had significantly more microglia and astrocytes in the ipsilateral hemisphere compared with the IS + GNF/GF group (*n* = 20). Scale bar: 100 μm. The numbers above each bar represent the sample size (*n*) for each group. The p values were determined by one-way ANOVA with the Bonferroni's post-hoc test in the three groups of contralateral and ipsilateral hemispheres and two-tailed independent samples t-test between the contralateral and ipsilateral hemispheres of the same group. All data are presented as the mean ± SEM. *p < 0.1, **p < 0.05, ***p < 0.01, ****p < 0.005.

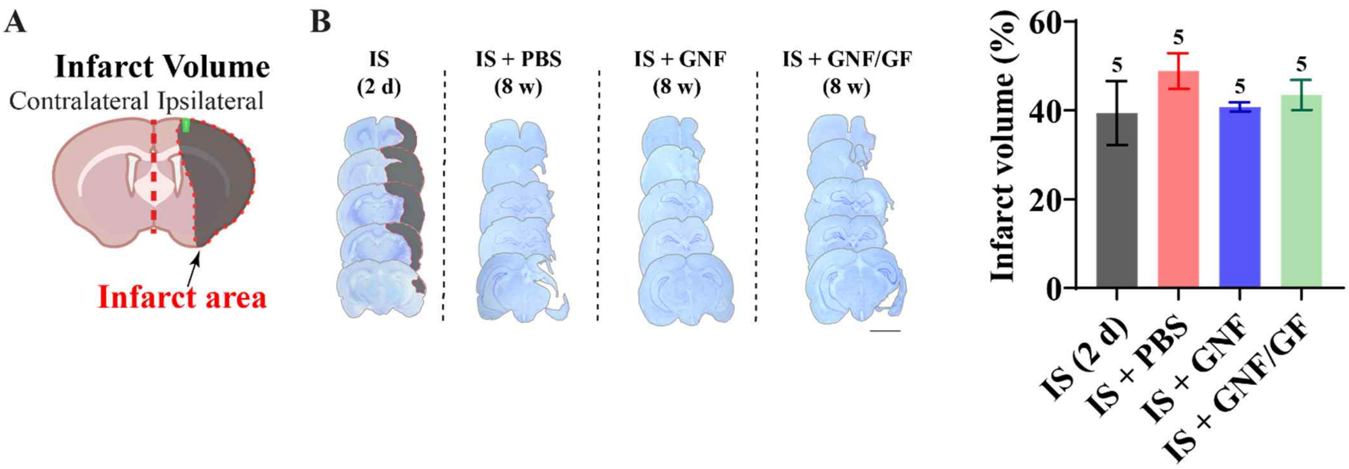


**Fig. S5. Analysis of infarct volume. (A)** Schematic of infarct area (grey area in the IS model) with the injection site marked in green. **(B) (left)** Representative cresyl violet stained images of sliced IS model brains 2 d after MCAO and 8 weeks after injection with PBS, GNF, or GNF/GF (infarct area highlighted in grey; scale bar: 5 mm) and **(right)** the measured infarct volume ($n$ = 5, p = 0.45). The number above each bar represents the sample size ($n$) for each group. The p values were determined by one-way ANOVA with a Bonferroni's post-hoc test in the four groups. All data are presented as the mean ± SEM. *p < 0.1, **p < 0.05, ***p < 0.01, ****p < 0.005.

**Table S1.** Modified neurological severity score (mNSS) details related to **Figs. 4B, 4C**, **S2**, and **S3** [52].

| Evaluation Factors | Points |
|---|---|
| **Motor Function Tests** | **6** |
| **Tail suspension** | **3** |
| Bending of the forelimb toward the body | 1 |
| Bending of the hindlimb toward the body | 1 |
| Head tilting >10° from the anteroposterior axis (> 30 s) | 1 |
| **Placing rat on the floor (normal=0; maximum=3)** | **3** |
| Walking normally | 0 |
| Difficulty walking in a straight line | 1 |
| Turning towards the side affected by paralysis | 2 |
| Falling towards the side affected by paralysis | 3 |
| **Sensory Function Tests** | **2** |
| Placing test (visual and tactile test) | 1 |
| Proprioceptive test (deep sensation, pushing the paw against the table edge to stimulate limb muscles) | 1 |
| **Beam Balance Tests** | **6** |
| Walking on a beam while maintaining a stable posture | 0 |
| Gripping the side of the beam | 1 |
| Holding onto the beam with one limb falling off | 2 |
| Clutching the beam with two limbs falling off, or spinning on the beam (> 60 s) | 3 |
| Maintains balance on the beam but falls (> 40 s) | 4 |
| Maintains balance but falls (> 20 s) | 5 |
| Falls off (< 20 s) | 6 |
| **Absent Reflexes and Abnormal Movements** | **4** |
| Lack of pinna reflex (no head movement when touching the external ear canal) | 1 |
| Lack of corneal reflex (no blink when the cornea is touched lightly with cotton) | 1 |
| No startle reflex (no motor response to a sudden noise, like snapping a clipboard) | 1 |
| Seizures, myodystony, myoclonus | 1 |
| **Maximum points** | **18** |

A score of 13–18 reflects severe injury, 7–12 indicates moderate injury, and 1–6 indicates mild injury.

**Table S2. Statistical summary of behavior tests. (A)** Two-way repeated measures ANOVA used to compare the data in **Fig S2. (B)** Two-way repeated-measures ANOVA used to compare the data in **Fig 4C. (C)** One-way ANOVA used to compare the data in **Fig S3. (D)** Two-way repeated-measures ANOVA used to compare the data in **Fig 4F**. All data are presented as the mean ± standard error of the mean.

**A**

| **Group (*n*)** | **mNSS** | | | | | | |
|---|---|---|---|---|---|---|---|
| | **- 3 d** | **- 1 d** | **Post-injection** | **1 w** | **2 w** | **3 w** | **4 w** |
| Ctrl + PBS (3) | 0.00 ± 0.00 | 0.00 ± 0.00 | 0.00 ± 0.00 | 0.33 ± 0.33 | 0.00 ± 0.00 | 0.33 ± 0.33 | 0.00 ± 0.00 |
| Ctrl + GNF (3) | 0.00 ± 0.00 | 0.00 ± 0.00 | 2.00 ± 1.15 | 2.33 ± 0.88 | 1.33 ± 0.67 | 0.67 ± 0.67 | 0.67 ± 0.67 |
| Ctrl + GNF/GF (3) | 0.00 ± 0.00 | 0.00 ± 0.00 | 2.67 ± 0.67 | 0.67 ± 0.67 | 0.33 ± 0.33 | 0.00 ± 0.00 | 0.00 ± 0.00 |
| Repeated measures ANOVA (time) | F (4, 24) = 4.047, p = 0.012 | | | | | | |
| Repeated measures ANOVA (time * Group) | F (8, 24) = 1.921, p = 0.103 | | | | | | |

**B**

| **Group (*n*)** | **mNSS** | | | | | | | | | |
|---|---|---|---|---|---|---|---|---|---|---|
| | **Post-IS** | **Post-injection** | **1 w** | **2 w** | **3 w** | **4 w** | **5 w** | **6 w** | **7 w** | **8 w** |
| IS + PBS (6) | 9.67 ± 0.92 | 8.00 ± 0.26 | 7.67 ± 0.67 | 7.17 ± 0.48 | 6.17 ± 0.91 | 5.67 ± 0.84 | 5.17 ± 0.87 | 5.00 ± 1.03 | 4.67 ± 0.84 | 5.00 ± 0.68 |
| IS + GNF (7) | 9.14 ± 0.34 | 7.29 ± 0.36 | 7.00 ± 0.38 | 5.43 ± 0.48 | 3.14 ± 0.63 | 2.86 ± 0.74 | 3.00 ± 0.53 | 2.71 ± 0.42 | 2.71 ± 0.52 | 2.29 ± 0.36 |
| IS + GNF/GF (7) | 8.29 ± 0.29 | 6.00 ± 0.49 | 5.57 ± 0.48 | 2.71 ± 0.42 | 2.00 ± 0.44 | 1.57 ± 0.48 | 1.57 ± 0.37 | 1.71 ± 0.47 | 1.29 ± 0.36 | 0.86 ± 0.46 |
| One-way ANOVA | F (2,17) = 1.613, p = 0.228 | F (2,17) = 6.594, p = 0.008 | F (2,17) = 4.434, p = 0.028 | F (2,17) = 23.407, p < 0.001 | F (2,17) = 10.068, p = 0.001 | F (2,17) = 8.806, p = 0.002 | F (2,17) = 8.805, p = 0.002 | F (2,17) = 6.279, p = 0.009 | F (2,17) = 8.237, p = 0.003 | F (2,17) = 16.983, p < 0.001 |
| Repeated measures ANOVA (time) | F (6.312, 107.31) = 56.403, p < 0.001 | | | | | | | | | |
| Repeated measures ANOVA (time * Group) | F (12.624, 107.31) = 1.918, p = 0.037 | | | | | | | | | |

**C**

| **Group (*n*)** | **mNSS (sensory)** | **mNSS (motor)** | **mNSS (balance)** | **mNSS (reflex)** |
|---|---|---|---|---|
| IS + PBS (6) | 1.33± 0.21 | 2.17 ± 0.31 | 1.50 ± 0.67 | 0.00 ± 0.00 |
| IS + GNF (7) | 0.14 ± 0.14 | 1.00 ± 0.00 | 0.71 ± 0.36 | 0.00 ± 0.00 |
| IS + GNF/GF (7) | 0.14 ± 0.14 | 0.71 ± 0.95 | 0.14 ± 0.14 | 0.00 ± 0.00 |
| One-way ANOVA | F (2,17) = 16.60, p < 0.001 | F (2,17) = 7.704, p = 0.004 | F (2,17) = 2.56, p = 0.107 | |

**D**

| **Group (*n*)** | **LH Swing Speed (cm/s)** | | | | | | | |
|---|---|---|---|---|---|---|---|---|
| | **Post-IS** | **Post-injection** | **1 w** | **2 w** | **3 w** | **4 w** | **7 w** | **8 w** |
| IS + PBS (6) | 50.31 ± 3.70 | 45.64 ± 7.42 | 46.53 ± 2.92 | 57.29 ± 3.89 | 61.42 ± 2.99 | 60.77 ± 8.11 | 63.13 ± 8.02 | 57.03 ± 11.15 |
| IS + GNF (7) | 53.00 ± 5.77 | 45.81 ± 2.52 | 48.90 ± 2.78 | 55.06 ± 5.04 | 49.57 ± 2.80 | 65.48 ± 3.71 | 57.29 ± 1.72 | 51.59 ± 2.84 |
| IS + GNF/GF (6) | 54.27 ± 1.28 | 63.54 ±7.01 | 58.80 ± 2.65 | 59.73 ± 3.46 | 69.70 ± 2.60 | 70.17 ± 2.70 | 71.78 ± 6.11 | 73.39 ± 9.33 |
| One-way ANOVA | F (2,9) = 0.252, p = 0.783 | F (2,16) = 7.277, p = 0.006 | F (2,16) = 14.91, p < 0.001 | F (2,16) = 0.314 p = 0.735 | F (2,16) = 5.732, p = 0.013 | F (2,16) = 1.772, p = 0.202 | F (2,16) = 2.909, p = 0.084 | F (2,16) = 3.331, p = 0.062 |
| Repeated measures ANOVA (time) | F (6, 96) = 3.760, p = 0.002 | | | | | | | |
| Repeated measures ANOVA (time * Group) | F (12, 96) = 0.981, p = 0.473 | | | | | | | |

**Table S3. Statistical summary of the rotarod test and fluorescence image analysis. (A)** One-way and two-way repeated measures ANOVA used to compare the data in **Fig 4I.** One-way ANOVA and two-tailed independent samples tests used to compare the data in **(B) Fig 5D, (C) Fig 5E**, **(D) Fig 5F**, and **(E) Fig S4**. All data are presented as the mean ± standard error of the mean.

**A**

| **Group (*n*)** | **Latency to falls (%)** | | | | | | | | | |
|---|---|---|---|---|---|---|---|---|---|---|
| | **Post-IS** | **Post-injection** | **1 w** | **2 w** | **3 w** | **4 w** | **5 w** | **6 w** | **7 w** | **8 w** |
| IS + PBS (6) | 48.54 ± 4.77 | 98.71 ± 23.64 | 94.61 ± 16.49 | 73.89 ± 9.49 | 75.94 ± 9.05 | 97.47 ± 11.22 | 89.37 ± 12.74 | 76.07 ± 11.59 | 82.86 ± 10.17 | 84.58 ± 15.25 |
| IS + GNF (7) | 51.39 ± 6.79 | 96.70 ± 15.18 | 101.47 ± 18.04 | 106.82 ± 5.62 | 117.00 ± 9.12 | 132.52 ± 20.40 | 119.77 ± 21.41 | 133.29 ± 23.03 | 122.34 ± 15.84 | 130.80 ± 23.89 |
| IS + GNF/GF (7) | 56.39 ± 7.36 | 106.47 ± 16.38 | 121.14 ± 20.47 | 142.88 ± 18.27 | 143.43 ± 23.05 | 118.92 ± 18.08 | 134.29 ± 19.38 | 116.23 ± 7.56 | 115.96 ± 5.41 | 114.39 ± 5.04 |
| One-way ANOVA | F (2,17) = 1.666, p = 0.218 | F (2,17) = 0.113, p = 0.894 | F (2,17) = 1.039, p = 0.375 | F (2,17) = 7.456, p = 0.005 | F (2,17) = 7.43, p = 0.005 | F (2,17) = 1.293, p = 0.302 | F (2,17) = 1.703, p = 0.214 | F (2,17) = 3.09, p = 0.075 | F (2,17) = 2.75, p = 0.099 | F (2,17) = 1.455, p = 0.239 |
| Repeated measures ANOVA (time) | F (4.027, 52.349) = 0.467, p = 0.761 | | | | | | | | | |
| Repeated measures ANOVA (time * Group) | F (8.054,52.349) = 1.411, p = 0.214 | | | | | | | | | |

**B**

| **Group (*n*)** | **# of lectin particle** | | **Independent sampled t** |
|---|---|---|---|
| | **Contralateral** | **Ipsilateral** | |
| IS + PBS (20) | 347.60 ± 25.36 | 284.55 ± 21.83 | t = 1.884, p = 0.067 |
| IS + GNF (22) | 379.50 ± 15.29 | 388.95 ± 11.29 | t = -0.498, p = 0.621 |
| IS + GNF/GF (18) | 360.56 ± 8.29 | 368.94 ± 12.94 | t = -0.546, p = 0.589 |
| One-way ANOVA | F (2, 57) = 0.827, p = 0.442 | F (2, 57) = 12.263, p < 0.001 | |

**C**

| **Group (*n*)** | **Average size of lectin (μm²)** | | **Independent sampled t** |
|---|---|---|---|
| | **Contralateral** | **Ipsilateral** | |
| IS + PBS (20) | 107.23 ± 5.59 | 85.19 ± 2.40 | t = 3.621, p = 0.001 |
| IS + GNF (22) | 123.74 ± 5.92 | 119.61 ± 4.81 | t = 0.541, p = 0.591 |
| IS + GNF/GF (18) | 130.95 ± 5.44 | 150.55 ± 12.08 | t = -1.479, p = 0.152 |
| One-way ANOVA | F (2, 57) = 4.386, p = 0.017 | F (2, 57) = 20.11, p < 0.001 | |

**D**

| **Group (*n*)** | **Lectin area fraction (%)** | | **Independent sampled t** |
|---|---|---|---|
| | **Contralateral** | **Ipsilateral** | |
| IS + PBS (20) | 3.06 ± 0.32 | 1.92 ± 0.16 | t = 3.174, p = 0.004 |
| IS + GNF (22) | 3.76 ± 0.24 | 3.71 ± 0.19 | t = 0.161, p = 0.873 |
| IS + GNF/GF (18) | 3.75 ± 0.18 | 4.39 ± 0.35 | t = -1.605, p = 0.121 |
| One-way ANOVA | F (2, 57) = 2.389, p = 0.101 | F (2, 57) = 28.045, p < 0.001 | |

**E**

| **Group (*n*)** | **# of Iba-1 positive cell** | | **Independent sampled t** |
|---|---|---|---|
| | **Contralateral** | **Ipsilateral** | |
| IS + PBS (19) | 13.63 ± 0.99 | 31.53 ± 2.76 | t = -6.102, p < 0.001 |
| IS + GNF (21) | 16.62 ± 2.28 | 34.10 ± 3.53 | t = -4.157, p < 0.001 |
| IS + GNF/GF (20) | 11.40 ± 1.02 | 21.45 ± 1.77 | t = 4.929, p < 0.001 |
| One-way ANOVA | F (2, 57) = 2.761, p = 0.072 | F (2, 57) = 5.70, p = 0.006 | |

| **Group (*n*)** | **# of GFAP positive cell** | | **Independent sampled t** |
|---|---|---|---|
| | **Contralateral** | **Ipsilateral** | |
| IS + PBS (17) | 21.00 ± 2.72 | 31.94 ± 2.41 | t = -3.012, p = 0.005 |
| IS + GNF (22) | 20.95 ± 1.48 | 35.91 ± 3.27 | t = -4.163, p < 0.001 |
| IS + GNF/GF (20) | 19.90 ± 1.73 | 22.25 ± 1.61 | t = -0.993, p = 0.327 |
| One-way ANOVA | F (2, 56) = 0.103, p = 0.903 | F (2, 56) = 7.583, p = 0.001 | |
